# Expression of concern: Detection of microplastics in human saphenous vein tissue using μFTIR: A pilot study

**DOI:** 10.1371/journal.pone.0320655

**Published:** 2025-03-24

**Authors:** The PLOS One Editors

The *PLOS One* Editors issue this Expression of Concern to notify readers that the raw spectra data underlying this study are no longer available. In the absence of these data, this article [1] is in breach of *PLOS One*’s Data Availability policy in place at the time this article was submitted.

After this article [1] was published, questions were asked about the spectra data underlying the article’s findings.

Specifically:

1. In Fig 1D, the sample spectra all have similar spectra profiles despite being identified as four different polymers.
2. Tissue samples are rich in fatty acids and proteinaceous material, and these organic materials are well-known, common false positives for plastic. Therefore, it is possible that residual fatty acids covered the particles and contributed to the composite infrared spectra, which could lead to misidentification, for example as alkyd resins.
3. The study confuses detection limit with spatial resolution.
4. Blank correction may not be recommended in some cases, such as when the size and polymer type of microplastics (MPs) in the blanks and samples are different, as reported in this study.

The corresponding author stated that the raw spectra data underlying the study are no longer available. They commented that tissue spectra are rarely an exact match with reference spectra and that this study was carried out at a time when the information about appropriate methods for the analysis of microplastic contaminants in human samples was limited. As a result, the research group adopted methodology commonly used in fish and mussel samples instead. The corresponding author agrees that a detection limit of 10 micron should have been used in this study, but commented that there is no standardized method for blank correction in microplastic research.

The concerns and the authors’ responses were reviewed by an independent member of the *PLOS One* Editorial Board who was involved in the original review of this article. They commented that the questions represent a current and ongoing debate in the analysis of microplastics from human and biota origin.

This article was also republished on March 5, 2025, to correct the author list and add James Hobkirk as the eighth author. Please download this article again to view the correct version. The originally published, uncorrected article and the republished, corrected article are provided here for reference.

## Supporting information

S1 File Originally published, uncorrected article. (PDF)

S2 File Republished, corrected article. (PDF)
