# Supplementary material for: Detection of microplastics in human saphenous vein tissue using μFTIR: A pilot study
Source: PLoS One. 2023 Feb 1;18(2):e0280594. doi: 10.1371/journal.pone.0280594 (PMC9891496; doi:10.1371/journal.pone.0280594)
Supplement: S1 Table — Additional columns present data used to determine Limit of Detection and Limit of Quantification values. Abbreviations; PVAc, poly (vinyl propionate/acetate); PVAE, polyvinyl acetate:Ethylene; PUR, polyurethane. (DOCX) [file pone.0280594.s001.docx]

**Supporting Information**

**S1 Table. Showing all identified polymers within vein samples and accounting for the same polymer if identified in controls.** Additional columns present data used to determine Limit of Detection and Limit of Quantification values. Abbreviations; PVAc, poly (vinyl propionate/acetate); PVAE, polyvinyl acetate:ethylene ; PUR, polyurethane.

| Polymer and patient sample ID | Polymer quantity in samples  **(A)** | Mean polymer quantity in controls ± SD  **(B)** | Blank correction A-B  **(C)** | Limit of Detection 3*SD of B OR 1.1/A (whichever is higher) **(LoD)** | Limit of Quantification 10*SD of B OR 3.3/A (whichever is higher) **(LoQ)** |
| --- | --- | --- | --- | --- | --- |
| **Alkyd Resin**  1  2  4  5 | 16  12  4  4 | 0±0 | 16  12  4  4 | 1.1  1.1  1.1  1.1 | 3.3  3.3  3.3  3.3 |
| **PVAc**  1  5 | 4  12 | 0±0 | 4  12 | 1.1  1.1 | 3.3  3.3 |
| **Nylon-Tie**  2  5 | 12  4 | 0±0 | 12  4 | 1.1  1.1 | 3.3  3.3 |
| **PVAE**  1 | 4 | 0±0 | 4 | 1.1 | 3.3 |
| **PUR**  2 | 8 | 0±0 | 8 | 1.1 | 3.3 |
