# Supplementary material for: Detection of microplastics in human saphenous vein tissue using μFTIR: A pilot study
Source: PLoS One. 2023 Feb 1;18(2):e0280594. doi: 10.1371/journal.pone.0280594 (PMC9891496; doi:10.1371/journal.pone.0280594)
Supplement: Attachment — Submitted filename: PLOS one REVISION2 response.docx [file pone.0280594.s002.docx]

**We thank the Editor and Reviewers for their additional time and attention in improving our manuscript. We address each point in turn below.**

Reviewer #1: The authors addressed most of the remarks in appropriate manner. Still a few remarks remain and had not been fully addressed by the authors.

**We thank the reviewer and make a commitment to fully address all comments.**

Major issues:
The authors did not include any assigned reference spectra of the Omnic Picta software to the spectra shown in Figure 1. For the reader the result cannot be followed in an easy manner for non-FTIR experts. This will even increase the impact of their manuscript.

**Figure 1 has been changed as requested. We have added reference spectra for 4 examples of particles – it now shows an image, the sample spectra and the reference spectra.**

Further, the authors state in their answers to the reviewer that the raw data of the FTIR spectra or measurement data was not saved or stored. Maybe I am misunderstood this part, but this is rather unusual for the various systems known to the reviewer, that the measurement files and related data were not stored. The reviewer acknowledges that the authors will consider a better data storage within their future work. Still, due to the embargo on the data on figshare, it was not possible to assess it for review to access it nor was it available as electronic supporting material for review only.

**We routinely take and store a screen shot of the spectra and the match result, plus a copy of the Omnic Picta software image of EVERY SINGLE particle analysed (including non-microplastics). The first two are shown on the operator screen panel together as shown below (in a typical example for explanation purposes only):**

**An Omnic Picta software image of the particle looks like this:**

**We also, occasionally, for example where we find a particle with a very high match % (such as >90%), will take a screen shot of the spectra only and store that for possible use in figures as indicative spectra:**

**It is possible to store the actual spectra file as raw data and to have the reference spectra added to the background of the image, using the Omnic Picta Software. This is the first time (since 2018) that a reviewer has asked us to upload all spectra in a data sharing resource. We agree that, in an ideal world, that data would all be collated and made available. Unfortunately, without knowing until recently (having seen chat on twitter) that some researchers use actual spectra data for meta-analysis, we only became aware of this recently. We routinely store word docs of collated screen shots (like those shown above), which we can see is not useful.**

**What we were trying to say is that, as *environmental biologists* using this analytical tool in an *applied way* to detect microplastics since 2018, we used the mFTIR equipment to analyse samples, only noting polymer type and % match initially (beginning 6 years ago). Then we evolved and stored collated word docs of image and spectra screen shots in more recent times, for our publications. That is what we meant by ‘evolving’ our methods and data storage. Many researchers publishing in microplastics field do not even use chemical characterisation at all, so we have been ahead of the majority in terms of robustness for that element. We also have been trail blazing with the LOD/LOQ approach in our most recent work. Given that we are not *analytical chemists*, we feel that we are working to quite high standards relative to most researchers publishing in this field. Thanks to kind people on Twitter, we are aware (in the past few months) that the specific spectra files are required for data meta-analysis. The colleagues who do use these datasets should be aware though, that it adds considerably more time to save them all than simply taking a screen shot and quick dump into a word file. Hopefully the reviewer has noticed that we had no significant funding for our work, we are not a large research team. Perhaps those scientists who use available datasets could help mentor/fund the smaller research teams so that they can reach a standard of data management that is the gold level, in the way that we have already evolved for LOD/LOQ.**

**We have uploaded the raw data excel spreadsheet and any sample spectra files that we had stored onto figshare – same link we supplied in the last revision. We removed the date embargo. The files are in PSA format and you will need the Omnic Picta or similar software to view them. We doubt that anyone would want to see all the word doc files containing every single screen dump of the spectra/match and images. Should anyone contact the corresponding author, we would be happy to share them. In the past 6 years, we have had a request for the excel type raw data (which we obliged), but not the spectra files so far. When (or if) we receive funding for future studies, we will factor in extra time for analysis and data management.**

Regarding the strong CO peak:
This is an important feature of all assigned spectra, so it also should be shortly highlighted in the discussion. It is still missing but will be important for future readers to contextualize the results.
**We added a sentence to the Discussion explaining the presence of the CO peak.**

Minor Issues:
Material and methods for FTIR: The current text is still not matching the recommended details of the Andrade et al. 2020 paper which can be found in the conclusions.

**We have copied and pasted the Andrade et al. 2020 paper conclusion suggested text below and we have underlined the elements we could find out and already had or have now added to the Methods section…**

*Measurements were made using a FTIR spectrometer (Model XXX, Commercial Brand), equipped with a [...] (Model and Brand –if needed- of the device used to measure, including detector and aperture when microspectrometry is used), operating in the AAA- BBB cm^−1^ mid-IR (or NIR) region, YY scans/sample, apodization ABC (type, check software), ZZ cm-1 nominal resolution. All spectra were corrected for light-reflectance penetration and baseline dis- placement. Background (empty and clean system) was done before [...] (e.g., measuring each sample). When needed, mention whether Kubelka-Munk or Kramers-Kronig corrections were applied; also specify spectra normalization, spectral treatment and other details that assure reproducibility of the measurements.*

**That highlighted above in yellow – we did not know what this is. The explanation in Andrade et al leads us to assume that we operated the equipment using whatever is the manufacturers standard setting for this parameter (since we have no idea how we would change it or why) and now report this in the methods section text.**

**That highlighted in green – we do say in the methods that no corrections/smoothing has been used.**

**That highlighted in light blue – not such facility on this model, so not applicable.**

Reviewer #2: An inter lab study would suffice for the data validation
FT Raman analysis to confirm microplastics in human saphenous vein tissue

**We looked at the previous publications for MPs in human tissues to date and can summarise the characterisation and any data validation if used here:**

- **Lung – microFTIR for characterisation, procedural blanks with LOD/LOQ.**
- **Blood – pyrolysis-GC-MS analysis for characterisation, blanks with LOD/LOQ.**
- **Colon – micro-FTIR and selected SEM, but reported no MPs in procedural blanks, no LOD/LOQ.**
- **Liver – micro-Raman for characterisation, procedural blanks with LOD/LOQ.**
- **Placenta – micro-Raman for characterisation, no procedural blanks with LOD/LOQ used.**
- **Breast milk – micro-Raman for characterisation, procedural blanks used but no LOD/LOQ used.**
- **Our study herein - microFTIR for characterisation, procedural blanks with LOD/LOQ.**

**We therefore respectfully decline to complete further validation analysis and wish to highlight that our study is in line with the other international published work on microplastics in human tissues, or (in some cases) surpasses it in terms of rigour.**
